# eNAMPT neutralization reduces preclinical ARDS severity via rectified NFkB and Akt/mTORC2 signaling

**DOI:** 10.1038/s41598-021-04444-9

**Published:** 2022-01-13

**Authors:** Tadeo Bermudez, Saad Sammani, Jin H. Song, Vivian Reyes Hernon, Carrie L. Kempf, Alexander N. Garcia, Jessica Burt, Matthew Hufford, Sara M. Camp, Anne E. Cress, Ankit A. Desai, Viswanathan Natarajan, Jeffrey R. Jacobson, Steven M. Dudek, Leopoldo C. Cancio, Julie Alvarez, Ruslan Rafikov, Yansong Li, Donna D. Zhang, Nancy G. Casanova, Christian Bime, Joe G. N. Garcia

**Affiliations:** 1 Department of Medicine, University of Arizona Health Sciences, Tucson, AZ USA; 2 Department of Radiation Oncology, University of Arizona Health Sciences, Tucson, AZ USA; 3 Department of Cellular and Molecular Medicine, University of Arizona Health Sciences, Tucson, AZ USA; 4 Department of Medicine, Indiana University, Indianapolis, IN USA; 5 Department of Medicine, University of Illinois Chicago, Chicago, IL USA; 6 Institute of Surgical Research, San Antonio, TX USA; 7 College of Pharmacy, University of Arizona Health Sciences, Tucson, AZ USA

**Keywords:** Inflammation, Preclinical research, Cytokines

## Abstract

Despite encouraging preclinical data, therapies to reduce ARDS mortality remains a globally unmet need, including during the COVID-19 pandemic. We previously identified extracellular nicotinamide phosphoribosyltransferase (eNAMPT) as a novel damage-associated molecular pattern protein (DAMP) via TLR4 ligation which regulates inflammatory cascade activation. eNAMPT is tightly linked to human ARDS by biomarker and genotyping studies in ARDS subjects. We now hypothesize that an eNAMPT-neutralizing mAb will significantly reduce the severity of ARDS lung inflammatory lung injury in diverse preclinical rat and porcine models. Sprague Dawley rats received eNAMPT mAb intravenously following exposure to intratracheal lipopolysaccharide (LPS) or to a traumatic blast (125 kPa) but prior to initiation of ventilator-induced lung injury (VILI) (4 h). Yucatan minipigs received intravenous eNAMPT mAb 2 h after initiation of septic shock and VILI (12 h). Each rat/porcine ARDS/VILI model was strongly associated with evidence of severe inflammatory lung injury with NFkB pathway activation and marked dysregulation of the Akt/mTORC2 signaling pathway. eNAMPT neutralization dramatically reduced inflammatory indices and the severity of lung injury in each rat/porcine ARDS/VILI model (~ 50% reduction) including reduction in serum lactate, and plasma levels of eNAMPT, IL-6, TNFα and Ang-2. The eNAMPT mAb further rectified NFkB pathway activation and preserved the Akt/mTORC2 signaling pathway. These results strongly support targeting the eNAMPT/TLR4 inflammatory pathway as a potential ARDS strategy to reduce inflammatory lung injury and ARDS mortality.

## Introduction

The Acute Respiratory Distress Syndrome (ARDS) is a vexing critical care illness occurring in response to sepsis, trauma, acid aspiration and both bacteria- and virus-induced pneumonias with extreme mortality rates^1^. The species-jumping coronaviruses, SARS-CoV, MERS-CoV and the SARS-CoV-2 producing the COVID-19 pandemic, are unprecedented causes of global ARDS fatalities^2^. COVID-19- and non-COVID-ARDS mortality is linked to multi-organ failure and mechanistically to unremitting lung and systemic inflammation^3^ generated by pathogen-activated innate immunity pathways (designed to contain the infection) and by damage-associated molecular pattern proteins DAMPs)-mediated amplification of inflammatory cascades elicited by exposure to mechanical ventilation, i.e. ventilator-induced lung injury (VILI)^1,3^. These evolutionarily-conserved inflammatory cascades produce massive increases in circulating inflammatory cytokines that result in vascular leak, multi-organ edema/dysfunction and ARDS/VILI death^3,4^.

The availability of approved SARS-CoV-2 vaccines and anti-viral SARS-CoV-2 drugs are of important clinical benefit but fail to address COVID-19 ARDS pathobiology with DAMP-mediated unchecked inflammation and multi-organ dysfunction via activation of evolutionarily-conserved inflammatory pathways^3,5^. Administration of the steroid, dexamethasone, appears to reduce mortality in severe COVID-19 ARDS subjects^4^, however, effective FDA–approved ARDS pharmacotherapies do not yet exist. Multiple promising ARDS therapies, supported by strong preclinical murine data, have ultimately failed in Phase 2/3 clinical trials^6–8^.

Utilizing genomic–intensive approaches and preclinical ARDS/VILI models^9–11^, we previously identified extracellular nicotinamide phosphoribosyltransferase (eNAMPT) as a novel DAMP and ARDS/VILI therapeutic target and biomarker^9–13^. eNAMPT expression is induced by ARDS-relevant stimuli (hypoxia, trauma, infection, ventilator stress)^14–17^ and similar to other DAMPs^5^, secreted eNAMPT activates a pathogen-recognition receptor, Toll–like receptor 4 (TLR4)^9^, to elicit profound NFkB-driven inflammatory processes involved in ARDS/VILI pathobiology^9^. We and others have successfully linked both elevated eNAMPT plasma levels^5,12^ and *NAMPT* single nucleotide polymorphisms (SNPs) to sepsis- and trauma-induced ARDS severity and mortality^13,16,18^. Importantly, preclinical murine ARDS/VILI studies demonstrated eNAMPT as a highly druggable target^10,11^.

The current study is designed to assess the research hypothesis that targeting the eNAMPT/TLR4 inflammatory cascade via an eNAMPT-neutralizing mAb will significantly reduce the severity of preclinical acute lung injury in rat and porcine ARDS/VILI models. These studies which have not been previously reported in either ARDS/VILI-challenged rats or pigs, attempt to maximize the translation of preclinical studies to successful human ARDS trials by extending prior murine results to a large animal model of ARDS/VILI. Our results in these multi-species ARDS/VILI models demonstrate that the intravenous delivery of an eNAMPT-neutralizing antibody dramatically reduces multiple indices of lung injury severity including histological injury, BAL inflammatory indices (protein, neutrophils), plasma IL-6 levels and dynamic and static lung compliance. Biochemical studies in LPS/VILI-exposed rat and porcine lung tissues revealed marked activation of the proinflammatory NFkB-mediated signaling pathway, increased production of reactive oxygen species (ROS), and severe dysregulation of the Akt/mTOR signaling pathway with each dysregulated pathway/protein rectified in animals receiving the intravenously-delivered eNAMPT-neutralizing mAb. These ARDS/VILI preclinical results strongly support the eNAMPT/TLR4 inflammatory pathway as viable and highly druggable therapeutic target. The combination of the eNAMPT-neutralizing mAb with predictive eNAMPT plasma biomarker and *NAMPT* genotyping assays provides an eNAMPT-focused personalized medicine platform to strategically identify high-risk subjects for ARDS clinical trials.

## Materials and methods

### Reagents

Reagents were purchased from Sigma-Aldrich (St. Louis, MO) unless otherwise noted. Antibodies for mTOR, Rictor, UCHL1, phospho-NF-kB p65, phospho-Akt^Ser473^ were purchased from Cell Signaling Technologies (Danvers, MA), β-actin and NF-kB from Invitrogen (Carlsbad, CA), goat anti-mouse IgG (Horseradish Peroxidase) from Jackson ImmunoResearch Laboratories. The anti-human goat NAMPT pAb was custom-generated as previously described^10,11^. The eNAMPT-neutralizing humanized mAb was provided by Aqualung Therapeutics (Tucson, AZ) as previously described^11^. See Supplemental Materials and Methods for more details.

### Animals studies

All animal care procedures, methods, animal randomization, and experiments were performed in accordance to relevant ARRIVE guidelines and regulations. In addition, all experiments were approved by and performed in accordance with relevant institutional licensing committee guidelines and regulations (Institutional Animal Care and Use Committee (IACUC), University of Arizona, protocol # 13-490). See Supplemental Materials and Methods for more details.

### LPS and LPS/VILI rat models of acute lung injury

Sprague Dawley male rats (300–350 g, Charles River, Wilmington, MA), anesthetized with IP ketamine (100 mg/kg)/xylazine (5 mg/kg), were intratracheally intubated for LPS instillation (*E. Coli* 0127: B8, 1 mg/kg) with harvesting 18 h post LPS exposure as reported^19^. For the “two-hit” model, anesthetized rats received LPS (0.1 mg/kg) and after 18 h were mechanically ventilated (4 h room air, Vt 20 ml/kg, RR 70 breaths/min, PEEP 0 cm H_2_O) (Advanced Ventilator System for Rodents, SAR-1000, CWE Incorporated, Ardmore, PA) as previously described^19,20^.

### Blast trauma/VILI rat model of acute lung injury

Anesthetized Sprague Dawley rats with carotid artery and jugular vein catheters were placed in prone position and received a blast overpressure exposure (BOP) of 150.4 kPa, using a compressed air-driven shock tube (Applied Research Associates, Littleton, CO). Followed a 30 min recovery period, rats were placed on mechanical ventilation for 4 h as above.

### Septic shock/VILI porcine model of acute lung injury

Male Yucatan minipigs (17–20 kg, S&S Farms, Ramona, California) were anesthetized with isoflurane followed by IV anesthesia (TIVA) with propofol (5–15 mg/kg/h), ketamine (2–6 mg/kg/h), and midazolam (0.25–0.75 mg/kg/h) and venous (auricular, dorsal pedal) and femoral artery catheters placed. Animals were ventilated on volume assist-control mode (Galileo mechanical ventilator, Hamilton Medical) and continuously monitored for mean arterial pressure, arterial blood gases, pH, pCO_2_, pO_2_, HCO_3_, SpO_2_, lactate, heart rate, RR, and end tidal carbon dioxide (ETCO_2_) (BM5 VET ICU monitoring system). Pigs received IV LPS (25 ug/kg) infused over a 2 h period while receiving 100% O_2_. The Vt was then increased from 13 to 20 ml/kg and the RR adjusted to maintain normal arterial blood gases (ABG) values. See Supplemental Table 1, and Supplemental Materials and Methods for more details.   

### Lung compliance

Lung compliance, a critical measure of lung stiffness dramatically affected by inflammatory lung injury, was measured hourly in ARDS-challenged pigs. Clinically, lung compliance is calculated by dividing lung volume by pressure yielding either static compliance i.e. the volume for any given applied pressure or dynamic compliance which is the compliance at any given time during the actual movement of air. Lung compliance is calculated by using the following equation, where ΔV is the change in volume, and ΔP is the change in pleural pressure. C = (ΔV)/ΔP. Static compliance (C stat) is the compliance during intervals without gas flow (inspiratory pause). It can be calculated by using this formula: C stat = Vt/(Pplat − PEEP) Where: = Static compliance, VT = Tidal volume. Pplat = Plateau pressure. Measured at the end of inhalation and prior to exhalation by using an inspiratory hold maneuver. PEEP = positive end-expiratory pressure. Dynamic compliance (C dyn) is the compliance during intervals of air flow (active inspiration). It can be calculated by using this formula: C dyn = Vt/(PIP − PEEP) Where: Cdyn = Dynamic compliance. VT = tidal volume. PIP = Peak inspiratory pressure (the maximum pressure during inspiration); PEEP = Positive End Expiratory Pressure.

### BAL analysis

Rat and porcine BAL fluids were obtained and processed for total protein measurements and BAL cell counts (PMNs) as we described^11,19,21^. Porcine BAL was performed using a disposable flexible bronchoscopy (Ambu®).

### Histology and immunohistochemistry analyses

Lungs fixed in 10% formalin underwent H&E and IHC staining (NAMPT expression) as previously described^9^. Histopathology images, 4 random lung fields/slide, 3 slides/rat or pig, were graded by a pathologist blinded to study groups. Images were selected for ImageJ software analysis of the area percentage of H&E and NAMPT staining^11^. See Supplemental Materials and Methods for more details.

### Biomarker measurements

A meso-scale ELISA platform (Meso Scale Diagnostics, Rockville, MD) was utilized for plasma measurements as previously described^11^.

### Biochemical analyses

Western blotting of lung tissue proteins was performed with densitometric analysis normalized to β-actin expression as previously reported^11^.

### Endothelial cell (EC) siRNA transfection

Human pulmonary artery endothelial cells (ECs) were cultured in essential growth medium (EGM-2) as described previously (Dudek et al., 2004). Small interfering RNAs (siRNAs) targeting UCHL1 (100 nM, GE Dharmacon, Lafayette, CO) or non-specific scrambled sequence were transfected with siPORT Amine (Ambion, Austin, TX) according to the manufacturer’s protocol and previously documented (Mitra et al., 2021). After 72 h of transfection, UCHL silencing in ECs was evaluated by Western blotting.

### Statistical analysis

Student t-test was used for comparisons between different groups with significance at *p* < 0.05. Statistical tests were performed using GraphPad Prism v7.0 (La Jolla, California).

## Results

### eNAMPT neutralization attenuates lung injury in the “one-hit” LPS rat model

**Figure 1:**
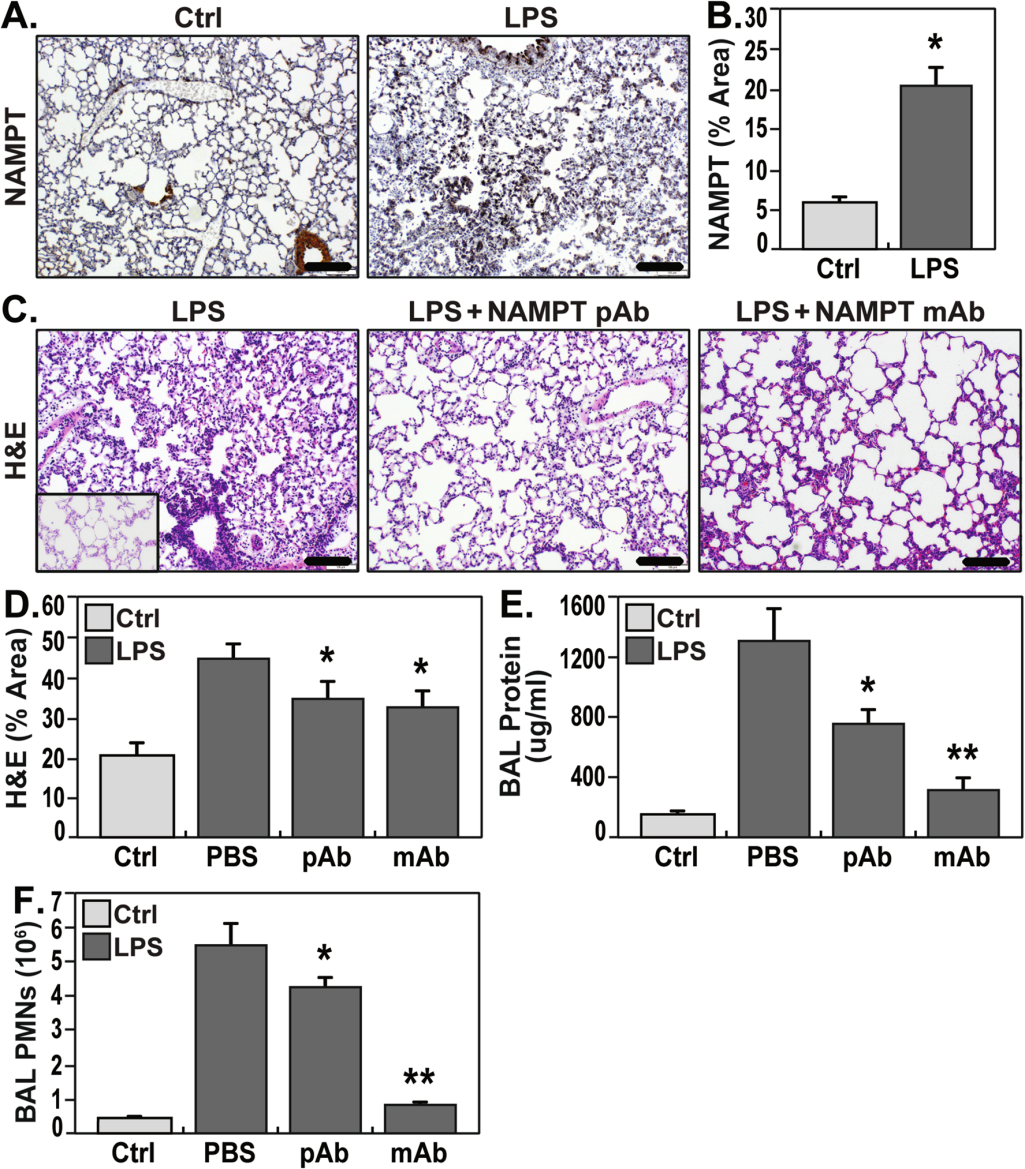
eNAMPT-neutralizing strategies attenuate rat lung injury in the preclinical “one-hit” LPS model. (**A**) IHC staining was performed to visualize NAMPT expression in lung tissues utilizing a rabbit anti-human mAb (Bethyl, Montgomery TX). Compared to control, unchallenged C57BL/6 J mice, marked increases in NAMPT expression were observed in lung tissues from Sprague Dawley rats LPS (18 h) exposed to the "one-hit" LPS ARDS model (intratracheal LPS, 1 mg/kg, 18 h) compared to controls. (**B**) Changes in NAMPT IHC staining with LPS exposure were quantitatively summarized by Image J software. *p < 0.01. (**C**, **D**) H&E lung tissue staining shows significant interstitial and alveolar inflammation with significant neutrophil infiltration and alveolar edema compared to control C57BL/6 J mice (inset). Histologic quantification of LPS-induced histologic injury showed significant reduction in lung injury severity in rats receiving either an IV-administered eNAMPT-neutralizing pAb (4 mg/kg, at time 0 with LPS injection) or the eNAMPT mAb (0.4 mg/kg, at time 0 with LPS injection) with (n =  > 5/group, *p < 0.05 for LPS-pAb or LPS-mAb vs. LPS-PBS control). (**E**, **F**) IV administration of either eNAMPT-neutralizing biologic intervention (pAb or mAb) significantly reduced LPS-induced increases in BAL protein (**E**) and BAL PMN counts (**F**) with superior efficacy of the eNAMPT mAb compared to the eNAMPT pAb (*p < 0.05 vs. LPS-PBS, **p < 0.05 mAb vs. pAb).

IHC staining of lung tissues from LPS-exposed rats demonstrated significantly increased NAMPT expression in alveolar epithelium, endothelium, macrophages, and parenchymal neutrophils (Fig. 1A) quantified by image analysis (Fig. 1B) (*p* < 0.05 compared to control rats). H&E tissue staining showed moderate alveolar inflammation with significant neutrophil infiltration and alveolar edema compared to control rats (inset) (Fig. 1C). Intravenous administration of either the eNAMPT-neutralizing pAb (4 mg/kg) or humanized mAb (0.4 mg/kg), given concurrently with LPS, significantly reduced histologic (Fig. 1C,D) and BAL evidence of inflammatory injury reflected by BAL protein levels and PMN counts (Fig. 1E,F) with the eNAMPT mAb providing superior protection compared to the eNAMPT pAb.

### eNAMPT neutralization attenuates lung injury in the “two-hit” LPS/VILI rat model

**Figure 2:**
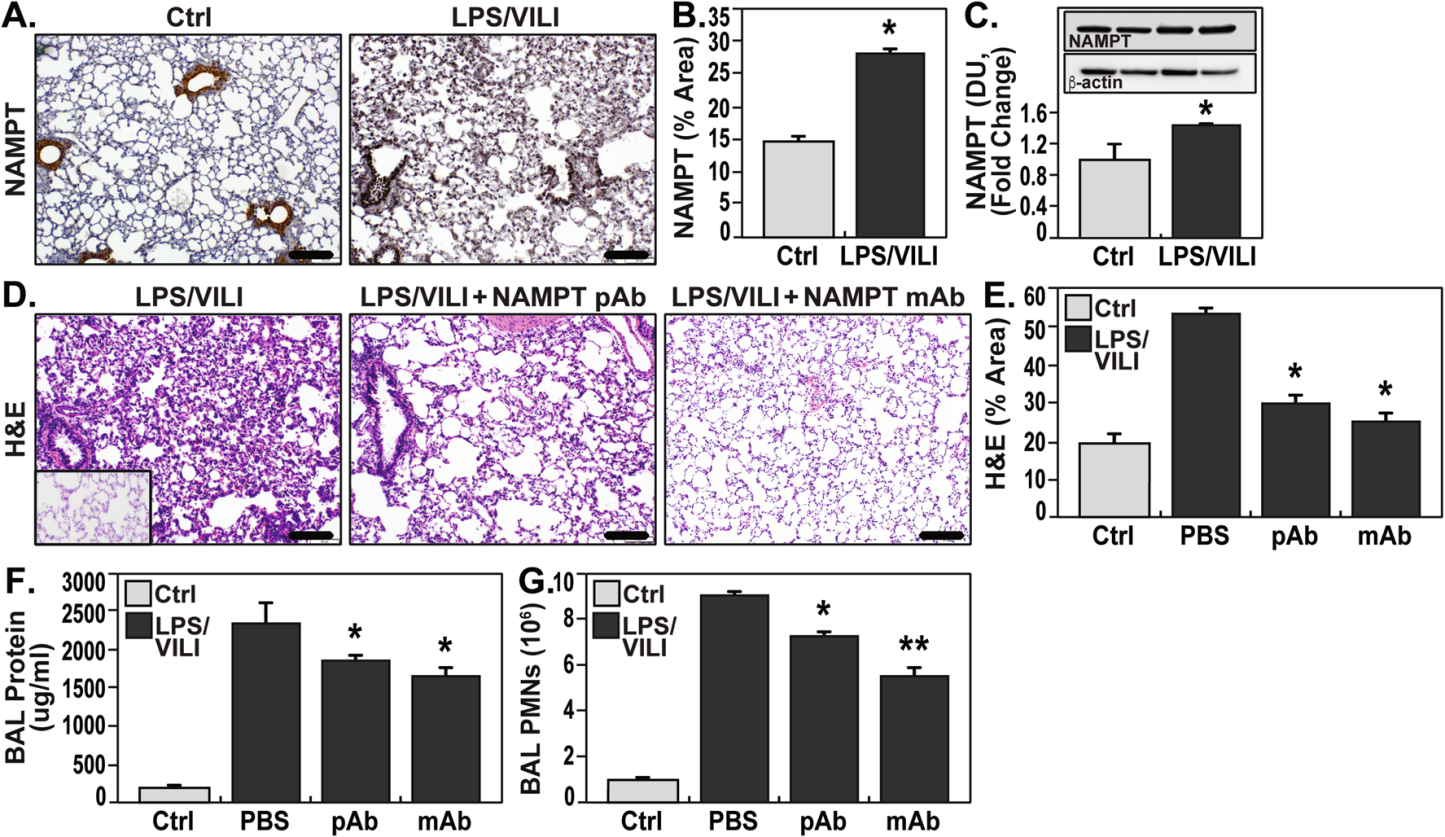
eNAMPT-neutralizing strategies attenuate rat lung injury in preclinical “two-hit” LPS/VILI model. (**A**) Increased lung tissue NAMPT staining (as in Fig. 1) in LPS/VILI -exposed rats compared to controls (LPS- 0.1 mg/kg, 18 h; VILI- 4 h tidal volume 20 mL/kg). (**B**) Image J IHC quantification of increased NAMPT expression with LPS/VILI exposure. *p < 0.01. (**C**) Western blot analysis shows significantly higher NAMPT expression in rat lung homogenates confirmed by densitometric analysis. *p < 0.05. (**D**, **E**) H&E lung tissue staining shows significant inflammatory injury with parenchymal neutrophil infiltration and both interstitial and alveolar edema which was significantly reduced by the eNAMPT-neutralizing pAb (4 mg/kg, at time 0 with LPS) and by the eNAMPT mAb (0.4 mg/kg, at time 0 with LPS) reflected by Image J Software. *p < 0.05 LPS-pAb/mAb versus LPS/VILI-PBS. (**F**, **G**) Infusion of either eNAMPT-neutralizing biologic intervention (pAb or mAb) in the “two-hit” ARDS/VILI rat model significantly reduced the marked increases in BAL protein content (**F**) and BAL PMN counts (**G**) (*p < 0.05 LPS-pAb/mAb vs. LPS/VILI-PBS; **p < 0.05 mAb vs. pAb).

Significant upregulation of NAMPT lung tissue expression was also observed in rats exposed to combined LPS and VILI compared to unexposed controls (Fig. 2A,B) (*p* < 0.05) and confirmed by NAMPT protein expression in lung homogenates (Fig. 2C). The severity of histologic inflammatory injury in LPS/VILI-challenged rats, with significant leukocyte infiltration and interstitial/alveolar edema, was greater than the “one-hit” ARDS model despite a tenfold lower LPS dose (Fig. 2D,E), likely reflecting the proinflammatory effects of exposure to mechanical ventilation. Similar to the “one-hit” model, LPS/VILI-induced inflammatory lung injury was significantly reduced in rats receiving either the eNAMPT pAb (4 mg/kg) or eNAMPT mAb (0.4 mg/kg), delivered intravenously concurrently with LPS and prior to exposure to mechanical ventilation (Fig. 2D,E). These results were highly consistent with the significant reductions in BAL protein/PMNs (Fig. 2F,G). The eNAMPT mAb again provided superior protection compared to the eNAMPT pAb (BAL PMNs).

### eNAMPT neutralization attenuates lung injury in the “two-hit” blast trauma/VILI rat model

**Figure 3:**
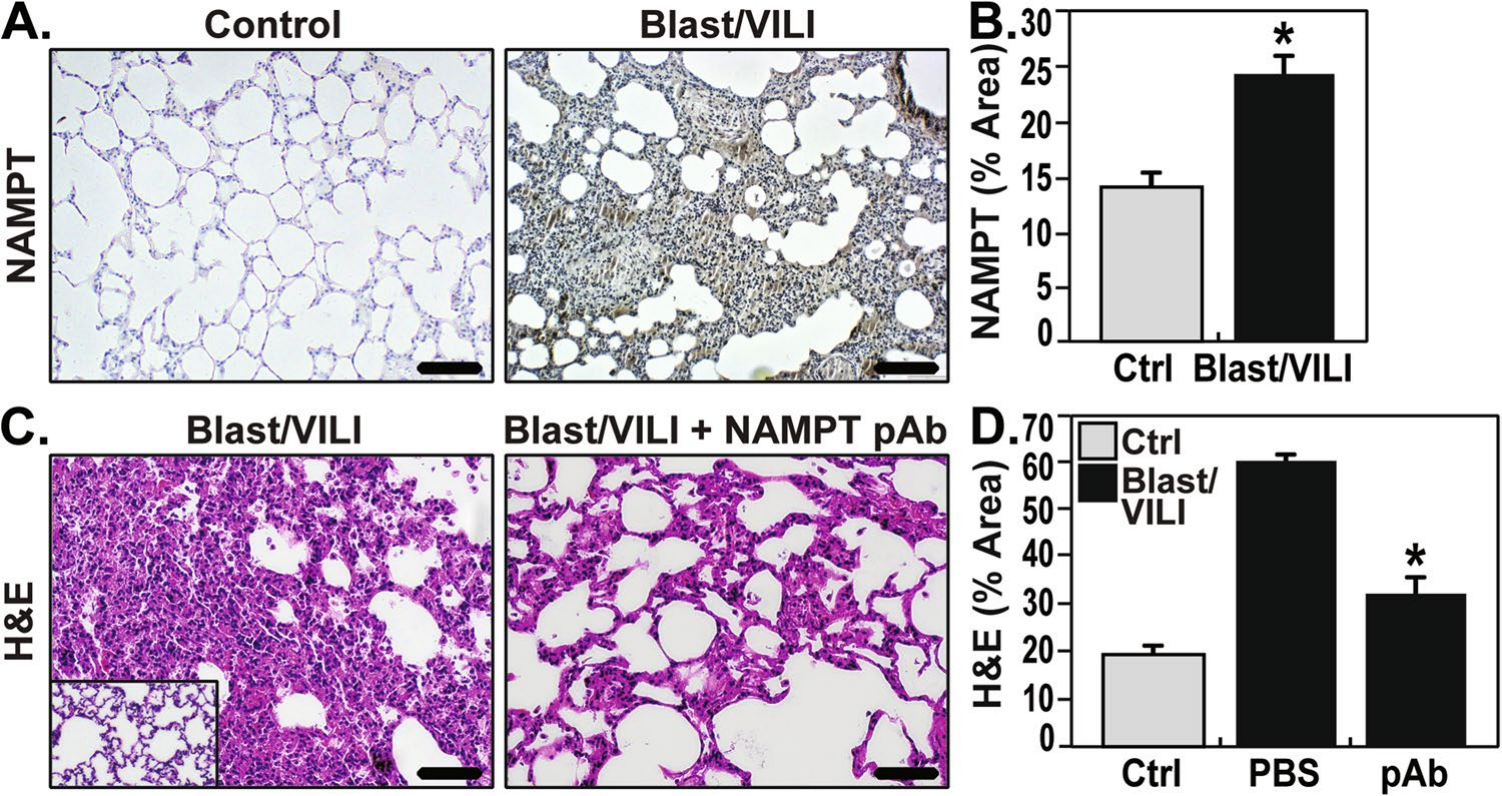
eNAMPT-neutralizing strategies attenuate rat lung injury in preclinical “two-hit” blast trauma/VILI. These studies were conducted at the US Army Institute of Surgical Research (Fort Sam Houston, San Antonio, TX). (**A**, **B**) Robust increases in NAMPT staining was observed in lung tissues (as in Fig. 1) from blast/VILI-exposed rats (BOP-150.4 kPa, t +  = 3.4 ms; VILI- 4 h, 70 breaths/min, tidal volume 10 ml/kg, 0 PEEP) with quantification by Image J software. *p < 0.05. (**C**, **D**) Microscopic H&E lung tissue staining in blast/VILI-challenged rats demonstrated profound inflammatory lung injury with increased PMN infiltration, alveolar septal thickening, edema, and hemorrhage (*p < 0.05 pAb Blast/VILI vs. untreated Blast/VILI) which was significantly reduced in rats receiving the eNAMPT-neutralizing pAb (4 mg/kg) delivered at time 0.5 h post blast exposure, but prior to mechanical ventilation.

Exposures to blunt or thoracic trauma are important contributors to ARDS prevalence, especially in warfighters^22^. A shock tube-generated single blast injury (150.4 kPa, 3.4 ms) followed by VILI exposure (4 h) was utilized to explore eNAMPT as a therapeutic target in a blast trauma/VILI rat model. NAMPT lung tissue expression was significantly increased in blast trauma/VILI-exposed rats (Fig. 3A,B). Macroscopic evaluation of H & E-stained lung tissues showed profound inflammatory lung injury with consolidation and edema confirmed by microscopic evidence of increased PMN infiltration, alveolar septal thickening, edema, and hemorrhage (Fig. 3C,D). The extent of blast trauma/VILI lung injury was significantly reduced by the eNAMPT-neutralizing pAb (4 mg/kg), delivered 30 min post blast, prior to VILI (Fig. 3C,D) (*p* < 0.01).

### eNAMPT neutralization attenuates lung injury in the “two-hit” septic shock/VILI porcine model

**Figure 4:**
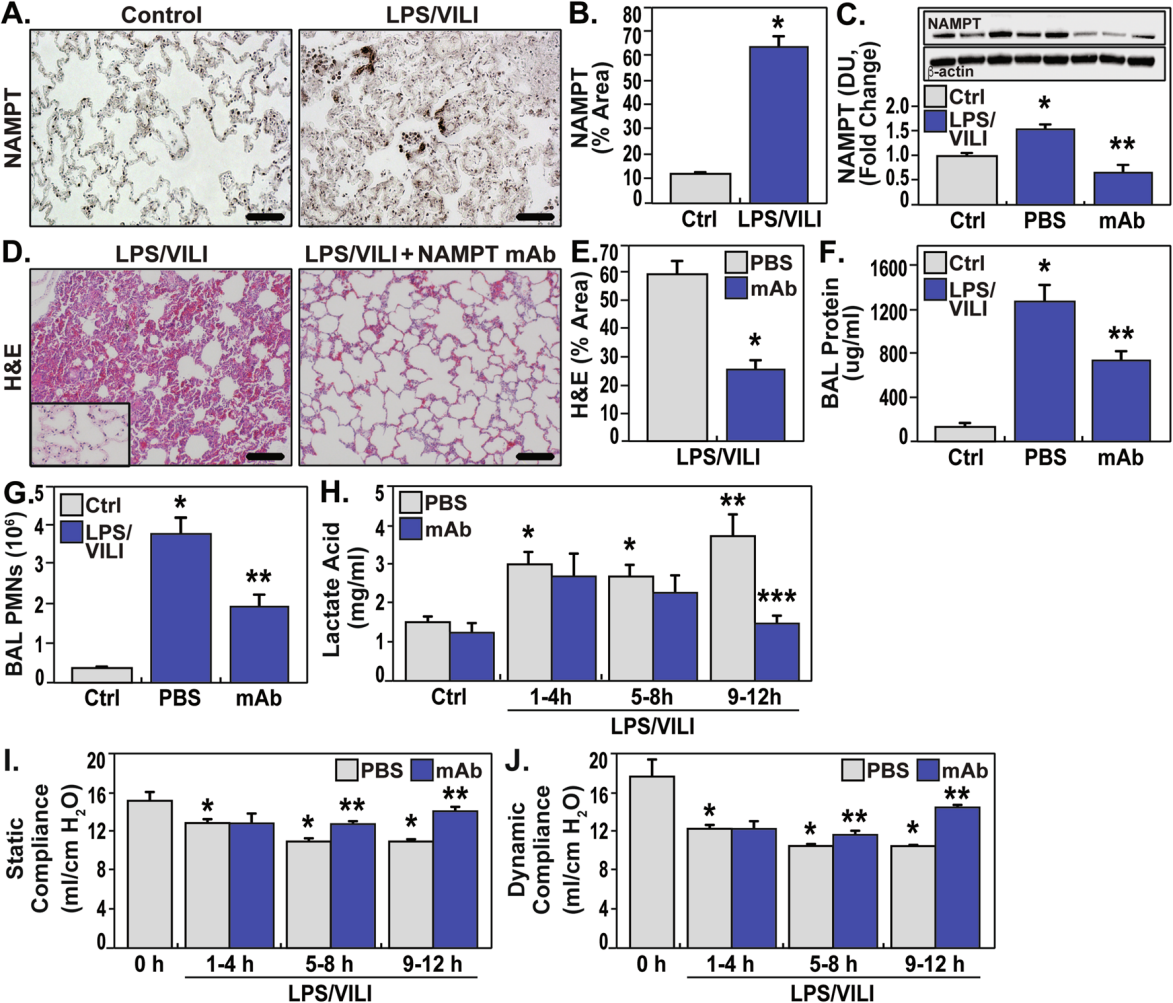
eNAMPT-neutralizing strategies attenuate porcine lung injury in preclinical “two-hit” septic shock/VILI. (**A**, **B**) Increased lung tissue NAMPT expression (as described in Fig. 1) in the “two-hit” preclinical porcine septic shock/VILI pigs compared to controls. *p < 0.001. (**C**) Western blot analysis confirmed significantly higher NAMPT expression in porcine septic shock/VILI pig lung homogenates (lanes 3–5) compared to controls (first 2 lanes). The eNAMPT-neutralizing mAb reduced NAMPT immunoreactivity in lung homogenates (lanes 6–8) (*p < 0.05 control vs. untreated LPS/VILI; **p < 0.05 mAb LPS/VILI vs. untreated LPS/VILI). (**D**–**G**) The eNAMPT mAb dramatically reduced H&E histologic and BAL evidence of severe inflammatory lung injury captured by quantification of injury (Image J Software) (**D**, **E**) and by reductions in BAL protein (**F**), and BAL total PMNs (**G**) (n = 6 pigs/group) (*p < 0.05 control vs. untreated LPS/VILI; **p < 0.05 mAb LPS/VILI vs. untreated LPS/VILI). (**H**) Serum lactic acid levels were obtained hourly and were significantly increased beginning 1 h after the onset of sepsis/VILI exposure. Lactate levels remained elevated each hour with a significant increase beginning at 9 h compared to 1–8 h. The humanized eNAMPT mAb significantly attenuated serum lactate increases over the 9–12 h time frame with values similar to control animals. (*p < 0.05 control vs. untreated LPS/VILI; **p < 0.05 untreated LPS/VILI 1–8 h vs. untreated LPS/VILI 9–12 h). ***p < 0.05 mAb LPS/VILI 9–12 h vs. untreated LPS/VILI 9–12 h). (**I**, **J**) Significant decreases in static and dynamic compliance, reflections of lung stiffness were observed in the initial 4 h period of exposure to sepsis/VILI**,** further decreasing and stabilizing through 9–12 h. Pigs receiving the eNAMPT neutralizing mAb at 2 h exhibited static and dynamic compliance values that were significantly and continuously improved over the 5–12 h period (*p* < 0.05) consistent with improved pulmonary/ventilatory physiologic indices. See Supplemental Table 1 and Supplemental Fig. 1.

To facilitate translation of preclinical results to human ARDS, Yucatan minipigs were exposed to both LPS-induced septic shock and high tidal volume ventilation with 100% FIO_2_ for 12 h. Immunohistochemical (Fig. 4A,B) and biochemical studies of lung homogenates (Fig. 4C) revealed significant septic shock/VILI-mediated increases in NAMPT lung tissue expression. Significant microscopic inflammatory lung injury was observed by H&E staining (Fig. 4D,E) and by increases in BAL protein/PMNs (Fig. 4F,G). The eNAMPT-neutralizing mAb delivered IV 2 h after the initiation of septic shock dramatically reduced NAMPT tissue expression (Fig. 4C) as well as provided a ~ 50% reduction in multiple indices of inflammatory lung injury (H&E staining, BAL protein/PMNs) (Fig. 4D,G). Serum lactate levels, a sensitive marker for septic shock, became significantly and consistently elevated as early as 1 h after LPS infusion with a further sharp rise 9–12 h post LPS/VILI exposure. This rise in serum lactate was abolished in pigs receiving the eNAMPT mAb as serum lactate levels returned to near baseline levels (Fig. 4H). Pigs receiving the human IgG_4_ (control, 0.4 mg/kg, n = 3) exhibited levels of histologic and BAL injury which were indistinguishable from pigs receiving IV-delivered PBS as placebo controls (data not shown).

### An eNAMPT-neutralizing mAb restores lung static and dynamic compliance in porcine LPS/VILI model

To determine if ALT-100 mAb-mediated reductions in lung water imbalance, as suggested by reduced BAL protein, translate to improved pulmonary/ventilatory physiologic indices, we performed hourly measurements of static and dynamic compliance, reflections of lung stiffness, which were bundled over 4-h periods for the entire 12 h period. These data indicate that static and dynamic compliance were significantly decreased in the initial 4 h period of exposure to sepsis/VILI (Fig. 4I, J) with further decreases at 5–8, remaining stable but reduced through 9–12 h of sepsis/VILI exposure. In contrast, pigs receiving the ALT-100 mAb at 2 h exhibited static and dynamic compliance values that were indistinguishable from untreated sepsis/VILI-exposed pigs in the initial 4 h period but significantly and continuously improved over the next 8 h (5–12 h) (*p* < 0.05).

### An eNAMPT-neutralizing mAb attenuates inflammatory cytokine increases in rat and porcine models of acute lung injury

**Figure 5:**
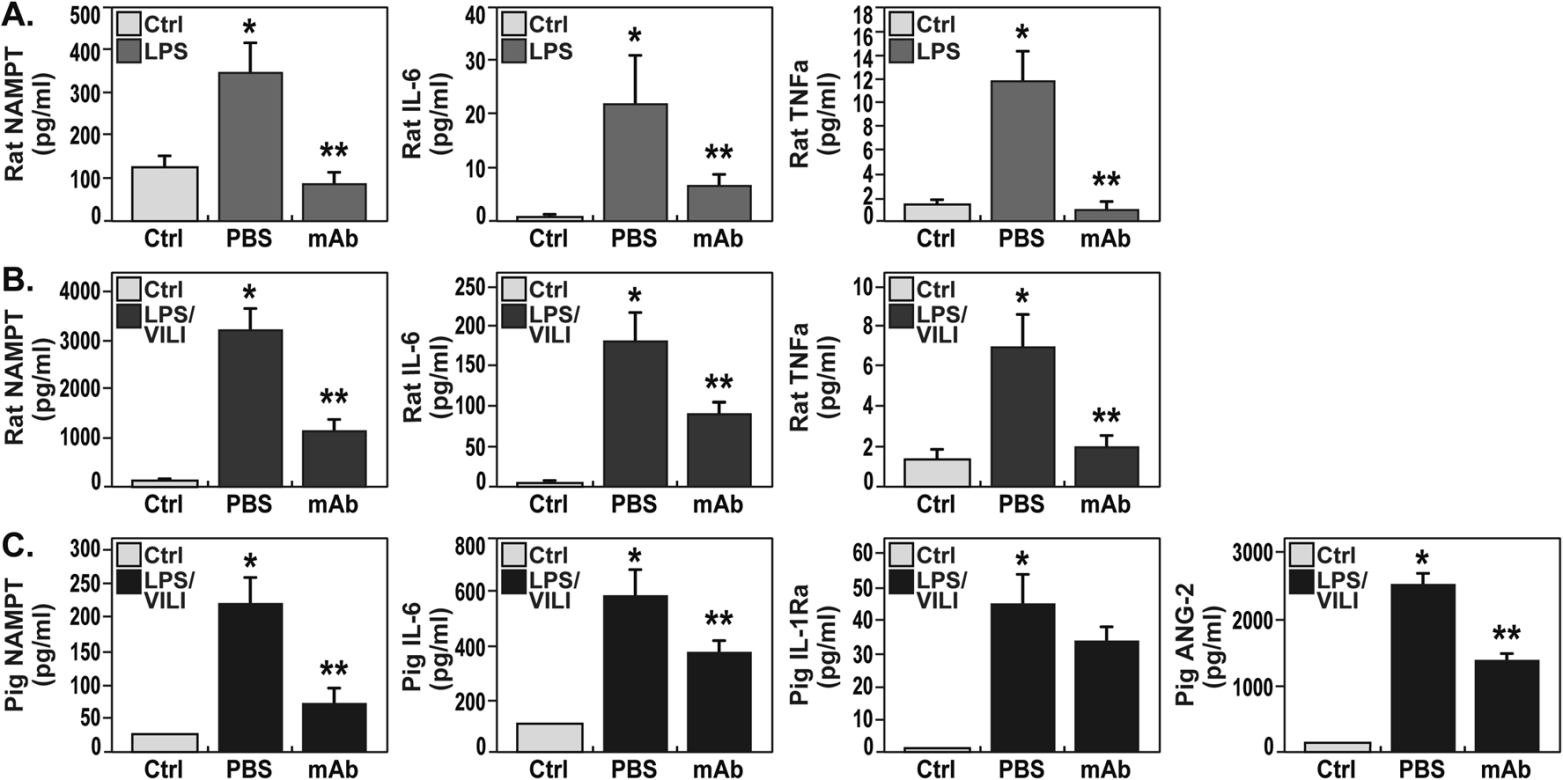
eNAMPT neutralization attenuates plasma biomarker increases in preclinical rat and porcine models of acute lung injury. (**A**) Plasma biomarker levels of eNAMPT, IL-6, and TNFα were measured (MesoScale Discovery platform) in control rats (n = 5) and at the conclusion of LPS-challenged rat experiments (18 h, n > 6 each group). The eNAMPT-neutralizing mAb markedly reduced plasma levels of each protein (*p < 0.05 control vs. untreated LPS; **p < 0.05 mAb LPS vs. untreated LPS). (**B**) Plasma biomarker levels of eNAMPT, IL-6 and TNFα measured in control rats (n = 5) and at the conclusion of ARDS/VILI-challenged rat experiments (n > 6 each group). The eNAMPT-neutralizing mAb markedly reduced plasma levels of each protein (*p < 0.05 control vs. untreated LPS/VILI; **p < 0.05 mAb LPS/VILI vs. untreated LPS/VILI). (**C**) Plasma levels of eNAMPT, IL-6, IL-1RA, and angiopoetin-2 were determined at time 0 and at 12 h in the porcine model of septic shock/VILI with significant increases in each protein at 12 h. With the exception of IL-1RA, the eNAMPT-neutralizing mAb markedly reduced LPS/VILI-induced elevations at 12 (*p < 0.05 control vs. untreated LPS/VILI; **p < 0.05 mAb LPS/VILI vs. untreated LPS/VILI).

Compared to acute lung injury-challenged rats or pigs receiving human IgG_4_ (control, 0.4 mg/kg, n = 3), animals receiving the eNAMPT-neutralizing mAb displayed markedly reduced plasma levels of eNAMPT, IL-6, and TNFα in both LPS- and LPS/VILI-exposed rat models (Fig. 5A,B) and reduced plasma levels of eNAMPT, IL-6, and angiopoetin-2 in LPS/VILI-exposed pigs (Fig. 5C). Plasma IL-1RA levels, while reduced, failed to reach statistical significance in LPS/VILI-exposed pigs receiving eNAMPTmAb.

### eNAMPT neutralization attenuates dysregulated inflammatory signaling in rat/porcine LPS/VILI models

**Figure 6:**
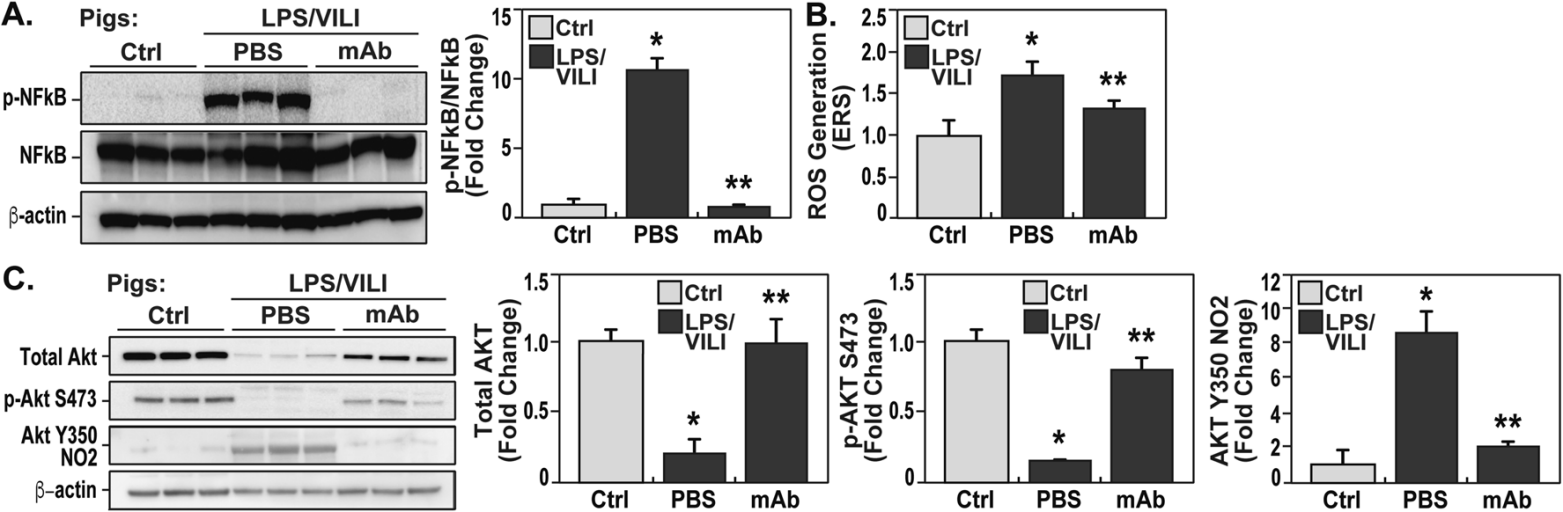
eNAMPT-neutralizing strategies attenuate ROS-, NFkB-, and Akt-dysregulated signaling in preclinical porcine LPS/VILI models. (**A**) Lung tissue homogenates were obtained from LPS/VILI-exposed untreated (n = 3) and eNAMPT mAb-treated Yucatan minipigs (n = 3) and compared to untreated control pigs (n = 3). Western blotting biochemical studies revealed striking increases in levels NFkB phosphorylation/total NFkB which abolished in pigs receiving the eNAMPT-neutralizing mAb. This was confirmed by densitometric evaluation of the ratio of p- NFkB/NFkB shown in the bar graph with eNAMPT mAb densitometric units comparable to untreated controls. (*p < 0.05 control vs. untreated LPS/VILI; **p < 0.05 mAb LPS/VILI vs. untreated LPS/VILI). (**B**) LPS/VILI-exposed pig lung tissue sections were utilized for reactive oxygen species (ROS) measurements measured using electron paramagnetic resonance spectroscopy (EPR). CMH signal is represented as nM/min/mg of lung tissue, and all treatment groups were normalized to controls. These studies demonstrated prominent increases in ROS burden which were markedly reduced in pigs receiving eNAMPT mAb treatment. (*p < 0.05 control vs. untreated LPS/VILI; **p < 0.05 mAb LPS/VILI vs. untreated LPS/VILI). (**C**) Evaluation of total Akt protein levels and Akt^473^ phosphorylation in LPS/VILI-exposed porcine lung homogenates (n = 3) demonstrated reductions in total Akt with concomitant loss of Akt^473^ phosphorylation. In addition, utilizing an Akt Y^350^ nitration antibody^26^, levels of Akt nitration were markedly increased in lung tissues from LPS/VILI-exposed Yucatan minipigs. Each LPS/VILI-induced Akt pathway alteration was reversed by treatment with the eNAMPT-neutralizing mAb including restoration of Akt, protein levels and Akt^S473^ phosphorylation in concert with near total abolishment of Akt^Y350^ nitration shown by densitometric assessment (*p < 0.05 control vs. untreated LPS/VILI; **p < 0.05 mAb LPS/VILI vs. untreated LPS/VILI).

Activation of evolutionarily-conserved, NFκB-inducible inflammatory cascades involve cytokine receptors and pattern-recognition receptors such as the TLR receptors^5^, that contribute to multi-organ failure and COVID-ARDS pathobiology/mortality^3^. Biochemical studies of LPS/VILI-exposed porcine lung tissue homogenates demonstrated striking increases in NFkB phosphorylation (Fig. 6A), reflecting strong activation of NFκB-induced signaling pathways. Excessive levels of reactive oxygen species (ROS) also contribute to the severity of inflammatory lung injury and sepsis/ARDS survival^23^. Electron spin resonance (EPR) measurements in LPS/VILI-exposed pig lung tissues confirmed significant increases in ROS burden (Fig. 6B). Both NFκB signaling pathway dysregulation and ROS generation were markedly reduced by delivery of the eNAMPT-neutralizing mAb (Fig. 6A,B), consistent with a critical role for the eNAMPT/TLR4 pathway in triggering LPS/VILI-induced activation of NFkB-driven inflammatory cascades and ROS generation.

**Figure 7:**
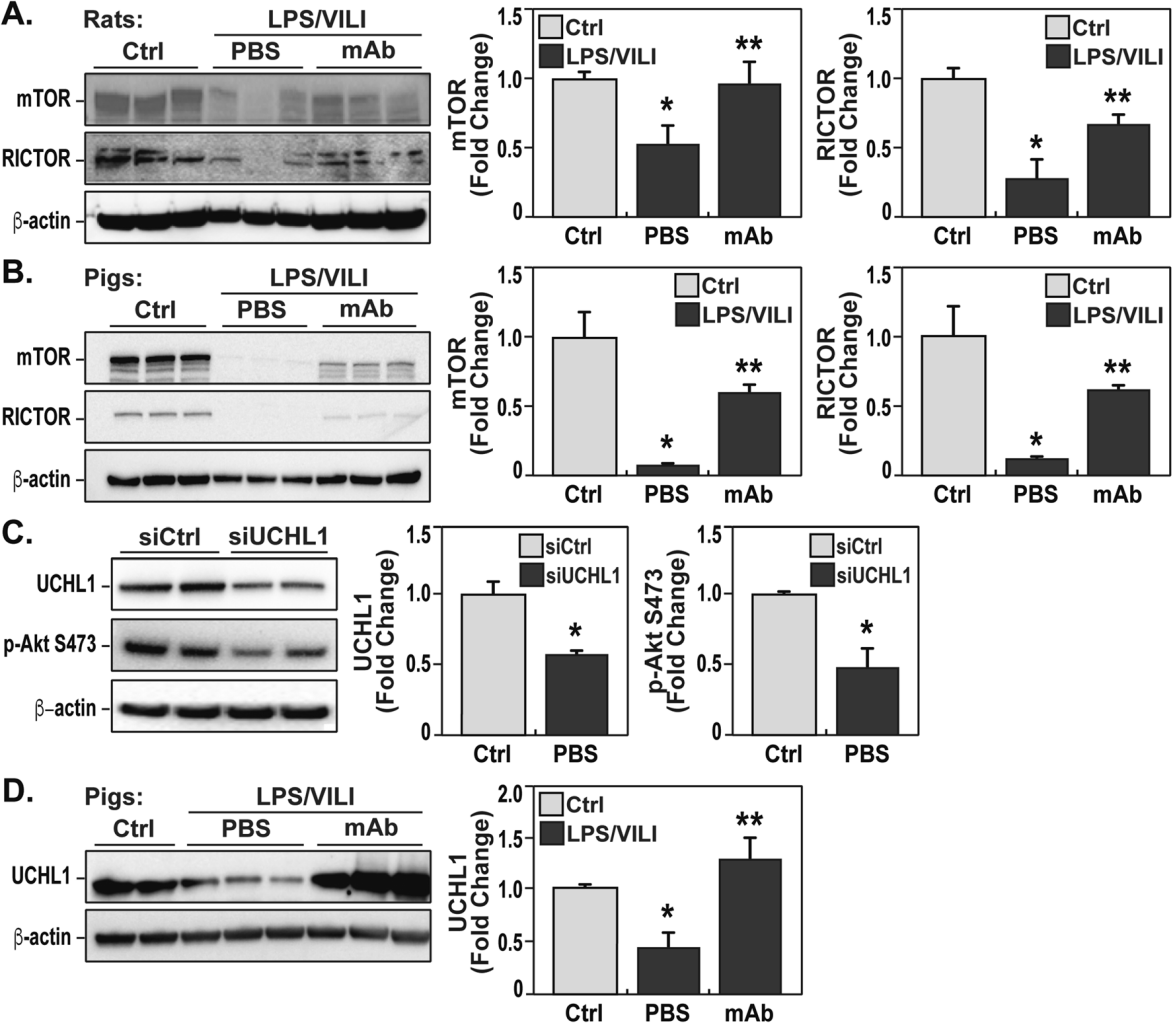
eNAMPT-neutralizing strategies preserves mTORC2 and UCHL1 protein expression in rat/porcine LPS/VILI models. (**A**, **B**) Evaluation of levels of total mTOR and the mTORC2 protein, Rictor, in LPS/VILI-exposed rat (n = 3) and porcine lung homogenates (n = 3) demonstrated marked reductions in total mTOR and Rictor protein expression which are significantly rectified by the eNAMPT-neutralizing mAb quantified by densitometric analysis (*p < 0.05 control vs. untreated LPS/VILI; **p < 0.05 mAb LPS/VILI vs. untreated LPS/VILI). (**C**) Human pulmonary artery endothelial cells (EC) were transfected with UCHL1 siRNA (100 nM) GE Dharmacon, Lafayette, CO) or non-specific scrambled sequence using transfection reagent siPORT (Ambion, Austin, TX) as previously described^9^. Cell lysates prepared 72 h post transfection for western blotting studies revealed reduced levels of UCHL1 and Akt^S473^ phosphorylation, quantified by densitometric analysis (*p < 0.05 control vs. UCHL1 siRNA). (**D**) UCHL1 protein expression was assessed in lung homogenates obtained from LPS/VILI-exposed Yucatan minipigs (n = 3) with and without treatment with the eNAMPT-neutralizing mAb. lung homogenates (n = 3) compared to control pig tissues. The LPS/VILI-mediated reduction in UCHL1 expression in lung tissues was restored to baseline levels by the eNAMPT mAb (*p < 0.05 control vs. untreated LPS/VILI; **p < 0.05 mAb LPS/VILI vs. untreated LPS/VILI).

The Akt-mTOR-Rictor signaling pathway is centrally involved in key homeostatic lung functions including preservation of vascular barrier integrity^24,25^ and is recognized as a dysregulated pathway in preclinical ARDS. We observed prominent alterations in Akt signaling in LPS/VILI-exposed porcine tissues (Fig. 6C) reflected by reduced protein levels of total Akt, loss of Akt^473^ phosphorylation, and striking increases in Akt Y^350^ nitration, a redox-sensitive post-translational modification that enhances Akt degradation^26^. In addition, LPS/VILI-exposed rats (Fig. 7A) and pigs (Fig. 7B) displayed reduced expression of mTORC2 kinase complex proteins, mTOR and Rictor, which regulate the Akt signaling pathway via Akt phosphorylation. Treatment with the eNAMPT-neutralizing mAb rectified Akt/mTOR pathway dysregulation with preservation of Akt, mTOR and Rictor protein levels, restoration of Akt^473^ phosphorylation levels and near-complete abolishment of Akt Y^350^ nitration (Figs. 6, 7). We have previously shown that Akt protein levels are regulated by protein ubiquitination involving the deubiquitinase, UCHL1 (ubiquitin carboxy-terminal hydrolase L1)^27^. Consistent with our prior reports^27^, Fig. 7C demonstrates that UCHL1 silencing in human lung ECs reduces Akt levels and Akt^473^ phosphorylation. Examination of UCHL1 protein expression in LPS/VILI-exposed porcine lung tissues revealed significant UCHL1 reduction with levels that were restored by the eNAMPT-neutralizing mAb (Fig. 7D). These results strongly suggest that reduced UCHL1 availability, evoked by LPS/VILI exposure, may contribute to Akt pathway dysregulation.

## Discussion

Our studies are highly consistent with hypothesis that ARDS-related multi-organ failure and mortality are directly influenced by evolutionarily-conserved inflammatory cascades triggered by bacterial/viral pathogens, trauma and ventilator-mediated mechanical stress involving the pathogen-receptor recognition TLR4 pathway^3,5,28^. Currently, no FDA-approved therapies address the unremitting ARDS-associated inflammation, a serious unmet need that has been dramatically highlighted by the COVID-19 pandemic^6–8,29^. Our studies, employing bench to beside translational studies^30^, an approach accelerated by the ongoing COVID-19 pandemic, have identified and validated a potential First in Human biologic therapy to reduce multi-organ failure and mortality in ARDS. eNAMPT is a novel innate immunity DAMP^9^ tightly linked to human ARDS via three complementary observations. First, both *NAMPT* transcriptional regulation and blood/lung protein expression, are highly induced by DAMP- and ARDS-relevant stimuli such as bacterial infection, hypoxia, shock, trauma and excessive ventilator-induced mechanical stress^14–17^ with plasma eNAMPT levels a biomarker for human ARDS severity^12,13^. Second, common *NAMPT* SNPs/haplotypes with > 5% minor allelic frequency in both non-Hispanic whites and vulnerable Black populations, confer significant risk for the development of ARDS and increased mortality^13,16,18^. Furthermore, ARDS-associated *NAMPT* SNPs influence promoter activity and eNAMPT plasma levels in ARDS subjects^16^. Lastly, our current mechanistic and therapeutic studies in diverse small and large preclinical models, indicate that circulating eNAMPT directly contributes to the pathobiology of ARDS/VILI via upstream TLR4 activation, a key innate immunity inflammatory cascade driving NFkB transcriptional activities and loss of lung vascular barrier integrity^9–11^. We greatly extend our prior murine studies highlighting eNAMPT involvement in acute lung injury^9–11,13^ by demonstrating a humanized eNAMPT-neutralizing mAb to dramatically reduce the severity of lung injury in four preclinical rat/porcine models of ARDS/VILI (~ 50% reduction) and to reduce lung water imbalances and improve respiratory compliance in ARDS/VILI-challenged pigs. Taken together, there is a substantial and compelling foundational basis for eNAMPT as a highly druggable therapeutic target in ARDS/VILI.

Our diverse small and large animal preclinical ARDS/VILI studies presented in this report directly address suggestions that a major failure of ARDS pharmacologic interventions is due to dependence on preclinical murine models^32^. However, we appreciate that the mere availability of a humanized eNAMPT mAb does not necessarily predict successful translation to successful human ARDS clinical trials. Factors such as the low resolution of the ARDS phenotype and the well-known heterogeneity of ARDS remain critical global challenges to successful ARDS therapeutic clinical trials conducted by ARDS clinical trial networks. We speculate that our preclinical study design to deliver the eNAMPT-neutralizing humanized mAb prior to initiation of mechanical ventilation, a useful clinical trial strategy whose feasibility is supported by recent clinical trials^33^, is predicted to attenuate or abolish the development of ventilator-mediated activation or amplification of inflammatory cascades, while minimizing the duration of mechanical ventilation and multi-organ injury, and improve ARDS survival. While the exact “golden hour” for delivery of the eNAMPT mAb is not yet definitively known, we are encouraged by results in our porcine septic shock/VILI model where dramatic reductions in lung injury were observed with delivery of the eNAMPT-neutralizing mAb 2 h after onset of septic shock/VILI.

Another unique feature of our work is the utilization of a blast trauma-induced rat lung injury model to test ARDS therapeutics. Trauma, either domestic or in military personnel surviving blast explosions, is a well-known cause for development of ARDS and we recently showed that both civilians and warfighters with traumatic injury exhibit marked blood eNAMPT elevations that track with the severity of traumatic injury^22^. The highly inflammatory and severely injurious nature of combined exposure to “shock tube”-delivered blast injury and VILI exposure, were significantly attenuated by eNAMPT pAb neutralization. Given that explosive or blast-induced injury is a significant life-threatening casualty in deployed U.S. Warfighters who are often subjected to delayed medical evacuation, the potential delivery of a “therapeutic” as well as a “preventive” strategy (eNAMPT mAb), administered within reasonable proximity to the time of the injury, may hold promise for military and domestic trauma victims with impending respiratory failure.

Mechanistically, the current study confirms an important role for activation of the TLR4-NFkB inflammatory cascade and ROS generation in the pathobiology of rat and porcine LPS/VILI. Striking increases in NFkB phosphorylation in LPS/VILI lung tissues were essentially abolished by the eNAMPT-neutralizing mAb, highlighting the key involvement of eNAMPT in LPS/VILI-induced TLR4 pathway activation, increases in ROS and subsequent systemic inflammation. Oxidative stress plays a significant role in ARDS pathogenesis with ROS-related gene expression significantly linked to survival in sepsis and ARDS^28^. We noted biochemical evidence of excess ROS in rat/porcine LPS/VILI models that clearly involve eNAMPT/TLR4 signaling given the attenuating effects of the eNAMPT-neutralizing mAb on ROS generation. Important mechanisms of ROS-mediated lung injury include activation of the lung endothelial cell (EC) contractile machinery to produce loss of barrier integrity, paracellular gaps and vascular permeability, essential components of ARDS pathobiology and mortality^3,28^. ROS also induces dysregulation of key homeostatic signaling pathways such as the Ser/Thr kinase Akt pathway and the Akt-regulatory, rapamycin-inhibitable mTOR-Rictor pathway, both pathways which contribute to the preservation of vascular integrity, a key determinant of ARDS severity. We noted that protein levels of Akt, mTOR and Rictor were each significantly reduced in LPS/VILI-exposed rat and porcine lung tissues with markedly diminished levels of Akt Ser^473^ phosphorylation, a post-translational modification catalyzed by the mTOR-Rictor complex that is involved in maintenance of lung vascular barrier integrity. In addition, and consistent with ROS involvement in Akt pathway dysregulation, marked increases in Akt Y^350^ nitration were observed in LPS/VILI-exposed lung tissues, an irreversible PTM that accelerates Akt degradation and has been observed in other lung disorders^26^. Importantly, each Akt/mTOR pathway alteration was prevented by delivery of the eNAMPT-neutralizing mAb. We have previously shown GADD45α (growth arrest, DNA damage-inducible protein) as a ROS-detoxifying ARDS target^34^ that modulates Akt signaling and EC barrier integrity via regulation of the expression on a deubiquitinase (DUB), UCHL1^27^. Reduced GADD45a or UCHL1 expression/DUB activity increases Akt ubiquitination and degradation and exacerbates inflammatory lung injury^27,35^. Our data indicate that Akt and UCHL1 protein levels are reduced in both UCHL1-silenced EC as well as in ARDS/VILI-challenged porcine lung tissues with UCHL1 levels preserved by treatment with the eNAMPT-neutralizing mAb.

In summary, we demonstrated the efficacy of an eNAMPT-neutralizing mAb as an ARDS therapeutic biologic utilizing diverse preclinical rat and porcine ARDS/VILI models to maximize successful preclinical translation to human ARDS. Our study suggests that the combined availability of: (i) an ARDS predictive plasma biomarker assay for eNAMPT^13^, either alone or as part of an ARDS biomarker panel^12^; (ii) a *NAMPT* genotype assay that identifies high-risk subjects^13,18^; and (iii) a highly efficacious eNAMPT-neutralizing humanized mAb^11^, may provide the opportunity to recruit high-risk ARDS subjects who are likely to respond to biologic targeting of the eNAMPT/TLR4 inflammatory pathway thus delivering personalized ICU medicine to address the unmet need for strategies to improve ARDS outcomes, a timely advance in the current COVID-19 pandemic landscape (Supplementary Information).

## Supplementary Information

Supplementary Information.

## Data Availability

No datasets were generated or analyzed during the current study.

## References

[1] Matthay MA (2019). Acute respiratory distress syndrome. Nat. Rev. Dis. Primers.

[2] Tzotzos SJ, Fischer B, Fischer H, Zeitlinger M (2020). Incidence of ARDS and outcomes in hospitalized patients with COVID-19: a global literature survey. Crit. Care.

[3] Bime C (2020). Strategies to DAMPen COVID-19-mediated lung and systemic inflammation and vascular injury. Transl. Res..

[4] Tomazini BM (2020). Effect of dexamethasone on days alive and ventilator-free in patients with moderate or severe acute respiratory distress syndrome and COVID-19: the CoDEX randomized clinical trial. JAMA.

[5] Gong T, Liu L, Jiang W, Zhou R (2020). DAMP-sensing receptors in sterile inflammation and inflammatory diseases. Nat. Rev. Immunol..

[6] JamaliMoghadamSiahkali S (2021). Safety and effectiveness of high-dose vitamin C in patients with COVID-19: a randomized open-label clinical trial. Eur. J. Med. Res..

[7] Soin AS (2021). Tocilizumab plus standard care versus standard care in patients in India with moderate to severe COVID-19-associated cytokine release syndrome (COVINTOC): an open-label, multicentre, randomised, controlled, phase 3 trial. Lancet Respir. Med..

[8] Stone JH (2020). Efficacy of tocilizumab in patients hospitalized with covid-19. N. Engl. J. Med..

[9] Camp SM (2015). Unique toll-like receptor 4 activation by NAMPT/PBEF induces NFkappaB signaling and inflammatory lung injury. Sci. Rep..

[10] Hong SB (2008). Essential role of pre-B-cell colony enhancing factor in ventilator-induced lung injury. Am. J. Respir. Crit. Care Med..

[11] Quijada H (2020). Endothelial eNAMPT amplifies preclinical acute lung injury: efficacy of an eNAMPT-neutralising mAb. Eur. Respir. J..

[12] Bime C (2019). Development of a biomarker mortality risk model in acute respiratory distress syndrome. Crit. Care.

[13] Ye SQ (2005). Pre-B-cell colony-enhancing factor as a potential novel biomarker in acute lung injury. Am. J. Respir. Crit. Care Med..

[14] Adyshev DM (2014). Mechanical stress induces pre-B-cell colony-enhancing factor/NAMPT expression via epigenetic regulation by miR-374a and miR-568 in human lung endothelium. Am. J. Respir. Cell Mol. Biol..

[15] Elangovan VR (2016). Endotoxin- and mechanical stress-induced epigenetic changes in the regulation of the nicotinamide phosphoribosyltransferase promoter. Pulm. Circ..

[16] Sun X (2014). The NAMPT promoter is regulated by mechanical stress, signal transducer and activator of transcription 5, and acute respiratory distress syndrome-associated genetic variants. Am. J. Respir. Cell Mol. Biol..

[17] Sun X (2020). Direct Extracellular NAMPT involvement in pulmonary hypertension and vascular remodeling. transcriptional regulation by SOX and HIF-2alpha. Am. J. Respir. Cell Mol. Biol..

[18] Bajwa EK, Yu CL, Gong MN, Thompson BT, Christiani DC (2007). Pre-B-cell colony-enhancing factor gene polymorphisms and risk of acute respiratory distress syndrome. Crit. Care Med..

[19] Nonas SA (2007). Use of consomic rats for genomic insights into ventilator-associated lung injury. Am. J. Physiol. Lung Cell Mol. Physiol..

[20] Goldman JL (2014). Pleiotropic effects of interleukin-6 in a "two-hit" murine model of acute respiratory distress syndrome. Pulm. Circ..

[21] Simon BA (2006). Microarray analysis of regional cellular responses to local mechanical stress in acute lung injury. Am. J. Physiol. Lung Cell Mol. Physiol..

[22] Recinos G (2009). ACS trauma centre designation and outcomes of post-traumatic ARDS: NTDB analysis and implications for trauma quality improvement. Injury.

[23] Bime C (2016). Reactive oxygen species-associated molecular signature predicts survival in patients with sepsis. Pulm. Circ..

[24] Singleton PA, Dudek SM, Ma SF, Garcia JG (2006). Transactivation of sphingosine 1-phosphate receptors is essential for vascular barrier regulation. Novel role for hyaluronan and CD44 receptor family. J. Biol. Chem..

[25] Zheng X, Zhang W, Hu X (2018). Different concentrations of lipopolysaccharide regulate barrier function through the PI3K/Akt signalling pathway in human pulmonary microvascular endothelial cells. Sci. Rep..

[26] Varghese MV (2020). Antioxidant-conjugated peptide attenuated metabolic reprogramming in pulmonary hypertension. Antioxidants (Basel).

[27] Mitra S (2011). Role of growth arrest and DNA damage-inducible alpha in Akt phosphorylation and ubiquitination after mechanical stress-induced vascular injury. Am. J. Respir. Crit. Care Med..

[28] Lynn H (2019). Genomic and genetic approaches to deciphering acute respiratory distress syndrome risk and mortality. Antioxid. Redox Signal..

[29] de Rivero Vaccari JC, Dietrich WD, Keane RW, de Rivero Vaccari JP (2020). The inflammasome in times of COVID-19. Front. Immunol..

[30] Eckle T (2013). HIF1A reduces acute lung injury by optimizing carbohydrate metabolism in the alveolar epithelium. PLoS Biol..

[31] ClinicalTrials.gov Identifier: NCT04478071. Vadadustat for the prevention and treatment of acute respiratory distress syndrome (ARDS) in hospitalized patients with coronavirus disease 2019 (COVID-19). (The University of Texas Health Science Center, 2020). https://www.clinicaltrials.gov/ct2/show/NCT04478071.

[32] Oakley C (2019). Ventilation following established ARDS: a preclinical model framework to improve predictive power. Thorax.

[33] National Heart L (2019). Early neuromuscular blockade in the acute respiratory distress syndrome. N. Engl. J. Med..

[34] Meyer NJ (2009). GADD45a is a novel candidate gene in inflammatory lung injury via influences on Akt signaling. FASEB J. Off. Publ. Fed. Am. Soc. Exp. Biol..

[35] Mitra S (2021). UCHL1, a deubiquitinating enzyme, regulates lung endothelial cell permeability in vitro and in vivo. Am. J. Physiol. Lung Cell Mol. Physiol..

