# Supplementary material for: eNAMPT neutralization reduces preclinical ARDS severity via rectified NFkB and Akt/mTORC2 signaling
Source: Sci Rep. 2022 Jan 13;12:696. doi: 10.1038/s41598-021-04444-9 (PMC8758770; doi:10.1038/s41598-021-04444-9)

## SUPPLEMENTAL MATERIALS AND METHODS

## eNAMPT neutralization reduces preclinical ARDS severity via rectified NFkB and Akt/mTORC2 signaling

**Reagents and antibodies.** All reagents including lipopolysaccharide (LPS, *E. Coli* 0127:B8 strain) were purchased from Sigma-Aldrich (St. Louis, MO) unless otherwise noted. Antibodies specific for mTOR (cat #2972S), Rictor (rabbit mAb 53A2, cat #2114S), UCHL1 (D3T2E Rabbit mAb, cat #13179S), phospho-NF-kB p65 (Ser536, 93H1 Rabbit mAb, cat #3033S), phospho-Akt (Thr308, rabbit mAb, 244F9, cat #4056S), phospho-Akt (Ser473) antibody duet (cat # 8200S) were all purchased from Cell Signaling Technologies (Danvers, MA). The β-actin Ab and NF-kB pAb (cat #PA5-16545) were obtained from Invitrogen (Carlsbad, CA). Human PBEF/Visfatin Biotinylated Antibody (cat #BAF4335) was purchased from R&D systems; Ultra Streptavidin-HRP (cat #N504) from ThermoFisher Scientific; Actin-HRP (cat #A3854-200UL) from Sigma-Aldrich. Peroxidase AffiniPure Goat Anti-Mouse IgG (H+L) (cat# 102646-170) and Anti-IgG (H+L) Goat Polyclonal Antibody (Horseradish Peroxidase) (cat #102645-188) were obtained from Jackson ImmunoResearch Laboratories. Akt tyrosine nitration (Y350 $NO_2$) antibody was generated as previously reported[24].

**Generation of eNAMPT-neutralizing polyclonal and humanized monoclonal antibodies.** The anti-human goat NAMPT polyclonal antibody (pAb) was custom-generated as we previously described[8 9] by Lampire Biological Laboratories, Inc. (Pipersville, PA) by immunizing goats against the full length rhNAMPT protein. The eNAMPT-neutralizing humanized mAb, ALT-100, was provided by Aqualung Therapeutics Corporation (Tucson, AZ) and was selected following *in*

*vitro* and *in vivo* screening of murine mAb-derived humanized variants as we have previously described[9].

**Endothelial cell (EC) siRNA transfection.** Human pulmonary artery endothelial cells (EC) were cultured in essential growth medium (EGM-2) containing 10% fetal bovine serum (Lonza, Walkersville, MD). Cells were placed in incubator at 37°C, 5% $CO_2$ and 95% humidity to achieve contact-inhibited monolayers as we previously described[9]. EC were transfected with siRNA (100 nM, GE Dharmacon, Lafayette, CO) specific for UCHL1 or non-specific scrambled sequence using transfection reagent siPORT *Amine* (Ambion, Austin, TX) in serum-free conditions according to the manufacturer's protocol. The medium was changed to EGM-2 containing 2% fetal bovine serum after 24 hrs of transfection and protein silencing was checked after 72 hrs of transfection. Silenced cells were utilized for western blotting studies described below.

**Animals utilized.** Sprague Dawley male rats (300-350 g) were purchased from Charles River (Wilmington, MA). For porcine experiments, Yucatan male minipigs (17-20 kg) were purchased from S&S Farms (Ramona, California). All rats and minipigs were housed under standard conditions (12 h light-dark cycle, 25-27 C$^o$, 40% humidity). Rats were kept in autoclaved microisolator cages or pens with free access to food and water throughout the duration of the experiments. All animal care procedures and experiments were approved by the Institutional Animal Care and Use Committee (University of Arizona).

**LPS "one-hit" preclinical ARDS rat model**. Sprague Dawley rats were anesthetized with a mixture of ketamine (100 mg/kg) and xylazine (5 mg/kg) (intraperitoneal injection). Animals were

intratracheally intubated with a 16-G angiocatheter which was used for LPS instillation (*E. Coli* 0127: B8, 1 mg/kg) and harvested 18 h later as we reported[17].

**LPS/VILI "two-hit" preclinical ARDS rat model**. Sprague Dawley male rats were anesthetized and LPS (0.1 mg/kg) intratracheally delivered as described in the "one-hit " LPS model. After 18 h, rats were re-anesthetized, intratracheally reintubated and connected to mechanical ventilation (Advanced Ventilator System for Rodents, SAR-1000, CWE Incorporated, Ardmore, PA) as previously described[17 18]. Supplemental ketamine and xylazine was provided to ensure adequate anesthetic depth during mechanical ventilation. Rats were ventilated for 4h with room air, tidal volume (Vt) 20 ml/kg, respiratory rate (RR) 70 breaths/min, and positive-end expiratory pressure (PEEP) 0 cm $H_2O$. Spontaneously breathing (SB) control animals received intratracheal PBS instead of LPS and following 18 h period, allowed to breathe spontaneously on room air for 4 h before harvesting.

**Blast trauma/VILI "two-hit" preclinical ARDS rat model**. Studies utilizing the rat model of blast trauma/VILI were conducted at the US Army Institute of Surgical Research (Fort Sam Houston, TX). Sprague Dawley male rats (10-12 weeks old, 350~475g) were anesthetized with a mixture of intraperitoneal ketamine (100 mg/kg) and xylazine (5 mg/kg) and isoflurane (1-3%) used to maintain anesthesia depth during mechanical ventilation. Carotid artery and jugular vein catheters were placed in anesthetized animals which were then placed in prone position with the head facing the blast front, and secured on a holder consisting of a flat plastic mesh suspended between two stainless steel rods, which run horizontally alongside the animal. A second plastic mesh was placed over the top of the rat to further secure the rat. The holder was attached to a swing

mechanism to allow the rat to partially recoil against the blast. Animals were then exposed to a blast overpressure [BOP = 150.4 kPa, duration of positive phase overpressure (t+) = 3.4 ms) using a compressed air-driven shock tube (Applied Research Associates, Littleton, CO). Rats were retrieved after blast exposure, observed for 30 min, then placed on mechanical ventilation for 4 hs (RR of 70 breaths/min, tidal volume (Vt) 10 ml/kg, 0 PEEP) using the VentElite small animal ventilator (Harvard Apparatus).

**Septic shock/VILI "two-hit" preclinical ARDS porcine model**. Male Yucatan minipigs were anesthetized with isoflurane to induce the anesthesia, followed by IV anesthesia (TIVA) with propofol (5-15mg/kg/h), ketamine (2-6mg/kg/h), and midazolam (0.25-0.75mg/kg/h) to maintain anesthesia during the 12h study. Two venous catheters were placed and secured into the auricular and dorsal pedal veins. The femoral artery was cannulated for mean arterial pressure (MAP) monitoring and hourly arterial blood sampling for arterial blood gases (ABG) (pH, pCO2, pO2, BEecf, HCO3, SpO2, and lactate), chemistries and electrolytes (I-Stat, CG4+, CHEM8+ cartridges). Animals were intratracheally intubated and connected to a Galileo mechanical ventilator (Hamilton Medical) on volume assist-control mode. Animals received continuous clinical monitoring for the entire 12-hr ICU duration: heart rate (HR), respiratory rate (RR), electrocardiogram, oxygen saturation ($SpO_2$), core temperature, end tidal carbon dioxide ($ETCO_2$) (BM5 VET ICU monitoring system). At the onset of the experiment, pigs received IV LPS (25ug/kg, *E. Coli* 0127-B8, Sigma Co) infused over a 2 hrs period while pigs received 100% $O_2$ and were ventilated with a tidal volume (Vt) of 13 ml/kg, RR of 15 breaths/min, and PEEP of 5 cm $H_2O$. After the 2 hrs LPS infusion, the Vt was increased to 20ml/kg, PEEP of 5 cm $H_2O$ and the RR adjusted to maintain ABG values within normal range. Bronchoalveolar lavage (BAL) was

performed using the disposable flexible bronchoscopy (Ambu®) at the beginning (baseline sample) and at 12 hrs study end. BAL samples were used to obtain BAL total and PMN cell counts as well as BAL protein levels.

**Delivery of the eNAMPT-neutralizing antibodies in rat and porcine ARDS/VILI preclinical models.** Intravenous eNAMPT-neutralizing pAb (4 mg/kg) or the eNAMPT mAb (0.4 mg/kg) was delivered concomitantly with LPS challenge in the "one-hit" LPS model, or with the "two-hit" LPS/VILI model. For the blast trauma/VILI "two-hit" rat model, specific groups of rats received intravenous eNAMPT pAb (4mg/kg) after blast exposure (0.5 h), prior to initiation of mechanical ventilation. For the porcine septic shock/VILI preclinical model, one group of pigs received either intravenous eNAMPT mAb (0.4 mg/kg) or PBS at the end of LPS infusion period, prior to high tidal volume ventilation exposure.

**Bronchoalveolar lavage (BAL) analysis**. At the termination of each experiment, rats were euthanized by approved IACUC methods (exsanguination after anesthesia). BAL was performed with 4 ml of cold Hank's buffered saline solution (HBSS) (Invitrogen) delivered intratracheally to the left lung after placing a surgical suture on the right lung followed by slow recovery of the BAL fluid as we have previously described[9 17 19]. In the porcine "two hit" model, BAL fluid collection was performed at two time points: at the beginning of the study prior to LPS instillation (baseline sample), and at the endpoint of study (12h). Cold Hank's buffered saline (HBSS, 10 ml) was instilled via a disposable flexible bronchoscopy (Ambu®). BAL fluid from both rat and porcine BALs were processed as we have described[9 17 19] with centrifugation (500*g*, 20 min, 4°C) and the pellets re-suspended in 200µL of cold HBSS. RBC Lysis Solution from Qiagen (1ml- 5min) was

used to eliminate RBC in the lavage. Samples were recentrifuged (500*g*, 10 min, 4°C) then pellets were re-suspended in 200µL of cold HBSS for total cell counting, using an automated cell counter (TC20; Bio-Rad, Hercules, CA) and for differential cell count to detect the number of PMNs by using cytospin centrifuge machine from Thermo Scientific (600 rpm, 25min) and Diff-Quick staining kit. The BAL supernatant was re-centrifuged (16,5000g, 10 min, 4°C), and the supernatant was collected for total protein measurements (Pierce BCA Protein Assay Kit, Thermo Scientific). BAL supernatant was stored at -80°C for further analysis.

**Quantitative lung histology and immunohistochemistry (IHC) analyses.** To assess alterations in the lung tissue morphology, lungs collected from sacrificed rats and pigs were fixed in 10% neutral buffered formalin for a minimum of 48 h, embedded in paraffin, sectioned, mounted onto slides, and stained with hematoxylin-eosin (H & E). Routine H&E slides were prepared using Richard-Allan hematoxylin, clarifier, bluing reagent and eosin as we have previously described[9]. The avidin-biotin-peroxidase method was utilized for IHC staining to visualize NAMPT expression in lung tissues (5 micron sections) (Bethyl Laboratories, Montgomery TX) or a rabbit IgG control (matched protein concentration, 1ug/ml, Vector Labs, Burlingame CA). Deparaffinized and rehydrated slides were ringed with an ImmunoPen rinsed in TBS, blocked for endogenous peroxidase using freshly prepared 0.5% hydrogen peroxide, 20 min, washed, protein block (Vector Labs) for 1 h, followed by avidin D and biotin block (Vector Labs), all at room temperature. Slides were incubated in primary or IgG isotype control, overnight at 4$^{o}$C with humidity. After washing, biotinylated secondary was applied for 1 h, washed and incubated with avidin-biotin complex (Vector Labs) 40 min, all at room temperature. The protein was visualized using DAB plus nickel (Vector Labs), 4 min, rinsed in tap water and counterstained with Mayers

hematoxylin (Newcomer Supply, Middleton, WI) 30 sec, washed in water, bluing reagent (Richard-Allan Scientific, San Diego CA) 10 sec, washed in water, dehydrated, cleared and cover-slipped with DPX.

Formalin-fixed paraffin embedded tissue sections were baked at 65°C overnight, washed in xylene 3 times, 7 minutes each, to remove paraffin, followed by passing through 100%, 75%, 50% isopropanol, and $_{dd}H20$ for rehydration. Antigen retrieval was performed using a sodium citrate buffer [10 mM sodium citrate, 0.05% Tween 20, pH 6.0] and heated at 97°C using decloaking chamber for 20 minutes. Slides were washed in washing buffer (0.1 M TRIS-HCl, 0.3 M NaCl, 0.1% Tween 20, and 7.7 uM NaN3, pH 7.6 at 25°C) followed by blocking buffer [5% (v/v) normal bovine serum, 0.1 M TRIS-HCl, and 0.15 M NaCl, pH 7.6 at 25°C] for 30 minutes. Primary antibodies were diluted in blocking buffer and incubated at 4°C overnight in a humidified chamber. Slides were washed 3 times in wash buffer and incubated with secondary antibody for 30 minutes to 1 hour at room temperature. Slides were washed 3 times in washing buffer and then mounted using ProLong Diamond Antifade Mountant (Thermo Fisher Scientific, P36970) and stored in the dark at room temperature overnight to cure the mountant.

Specimens were imaged using Zeiss Axiovert Photomicroscope using a 10X objective (NA 0.4). Histological images from each group captured with light microscopy (Olympus digital camera) at 10x magnification, were randomly selected for quantification of H&E and NAMPT staining using ImageJ software[9] (different sections of each slide). For H&E image analysis, the percentage of area selected for measurement with all images processed and stored for statistical analysis. For NAMPT staining image analysis, color segmentation plugin was utilized with POINTCROSS tool applied to each NAMPT staining image with a total of 3 color clusters. The

area percentage of each color cluster was recorded and saved for statistical analysis as we have reported[9].

**Plasma and BAL biomarker measurements.** A meso-scale ELISA platform (Meso Scale Diagnostics, Rockville, MD) was utilized as we previously described[9] for measurements of plasma levels of eNAMPT, IL-6 and TNFa in rats and eNAMPT, IL-6, IL-1RA and angiopoetin-2 in pigs. Each biotinylated antibody (10 µg/ml, specific for each analyte, was mixed with a different linker for each analyte and incubated for 30 min at 250C. The reaction was terminated with 200 µl of free biotin solution and 600 µl of the 10x U-PLEX linked biotinylated antibody solution with 50 µl of coating solution was added to each well in 96 well plate and incubated for 1 h (800 rpm shaking, 250C). After washing, each well was supplemented with 25 µl of diluent and 25 µl of calibrator or samples/standards, incubated for 1 h (800 rpm shaking, 250C). After washing (TBS-T), each well was supplemented with 50 µl/well of 1x detection antibody solution, again incubated for 1h, washed and supplemented with 2x Read Buffer T followed by plate imaging and calculation of the absolute concentration values based on standards[9].

**Western blotting and biochemical tissue analyses.** Western blotting of proteins within rat and porcine lung tissue homogenates was performed as we have previously reported[9]. Snap frozen lung tissues were homogenized in RIPA buffer (50 mmol/L Tris-HCl pH 7.4, 150 mmol/L NaCl, 0.5 % sodium deoxycholate, 0.1 % SDS, 1% NP-40, 5 mmol/L EDTA) supplemented with complete protease/phosphatase inhibitor cocktail (Cell Signaling Cat #5872S) using tissue grinder with glass pestles (VWR Cat #26307-606). After centrifugation (15,000 g for 20 min at 4°C), protein concentration of homogenates was determined by Bio-Rad DC protein assay (cat #5000112).

Following incubation 5 min at 90°C in loading buffer, aliquots containing equal amounts of protein (25–30 ug) were subjected to sodium dodecyl sulfate polyacrylamide gel electrophoresis (SDS-PAGE). Subsequently, proteins were transferred to PVDF membranes and probed with specific primary antibodies by horseradish peroxidase-conjugated secondary antibodies. Proteins were visualized using an ECL system (Pierce West Pico cat #34580) and ChemiDoc MP imaging system (Bio-Rad). Densitometric analysis was performed using Bio-Rad Image Lab 6.01 software by normalizing the levels of proteins to b-actin expression. The levels of phosphor-proteins were quantified by normalizing the levels to their respective total proteins[9].

**Reactive oxygen species (ROS) measurements in porcine lung tissue:** The generation of reactive oxygen species was measured using electron paramagnetic resonance spectroscopy (EPR). Briefly, ~50-100 mg of lung tissue was incubated with 20 mM Krebs-HEPES buffer (pH 7.4) containing 200 μM CMH for 30 min. Following treatment, buffer was collected, and changes in CMH oxidation were measured for 15 min using the e-scan Multipurpose Bench-top EPR system (Noxygen Science and Transfer Diagnostics GmbH). CMH signal is represented as nM/min/mg of lung tissue, and all treatment groups were normalized to controls.

**Statistical analysis.** Continuous data were compared using nonparametric methods and categorical data by chi square test. Where applicable, standard one-way ANOVA was used and groups were compared using the Newman-Keuls test. Differences between groups were considered statistically significant when p values were less than 0.05 ($p < 0.05$). Two-way ANOVA was used to compare the means of data from two or more different experimental groups. If significant differences were present by ANOVA ($p < 0.05$), a least significant differences (LSD) test was

performed post hoc. Between group differences were considered statistically significant when $p < 0.05$. Statistical tests were performed using GraphPad Prism version 7.00 for Windows, GraphPad Software, La Jolla California USA, www.graphpad.com.

## SUPPLEMENTAL TABLES AND FIGURES

**Supplemental Table 1. Hemodynamic parameters in the porcine ARDS models.**

| | Baseline | 6h | | 12h | |
|---|---|---|---|---|---|
| | **N=12** | **ARDS n=6** | **ARDS Ab n=6** | **ARDS n=6** | **ARDS Ab n=6** |
| **MAP** | 78.6 ± 2.5 | 89.2 ± 8.0 | 89 ± 2.8 | 77 ± 9.7 | 80.6 ± 0.3 |
| **HR** | 110 ± 2.4 | 116 ± 8.3 | 118 ± 3 | 123 ± 1.8 | 111 ± 7 |
| **RR** | 16 ± 0.9 | 11.8 ± 0.48 | 12.3 ± 0.3 | 11.3 ± 0.5 | 12 ± 0 |
| **PCO2** | 39.3 ± 1.8 | 31.1 ± 0.5 | 32.6 ± 4.4 | 30.1 ± 1.3 | 33.4 ± 1.9 |
| **SaO2** | 98.8 ± 0.22 | 98.6 ± 0.5 | 97.6 ± 0.6 | 99 ± 0.3 | 99 ± 0 |
| **PH** | 7.41 ± 0.01 | 7.28 ± 0.12 | 7.3 ± 0.06 | 7.35 ± 0.01 | 7.25 ± 0.03 |
| **HCO3** | 25.5 ± 0.7 | 18.78 ± 1.4 | 17.1 ± 0.49 | 17.1 ± 0.5 | 15.7 ± 0.36 |
| **Lactic acid** | 1.8 ± 0.27 | 3.87 ± 1.1 | 2.82 ± 0.5* | 3.94 ± 0.9 | 1.65 0.24* |

*P<0.05 vs no mAb

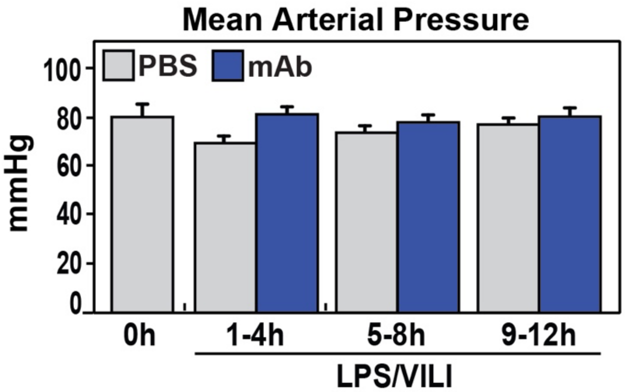


**Supplemental Figure 1.** The femoral artery was cannulated for mean arterial pressure (MAP) monitoring. Animals received continuous clinical monitoring for the entire 12-hr ICU duration: MAP was maintained within physiological values.

Figure 2C

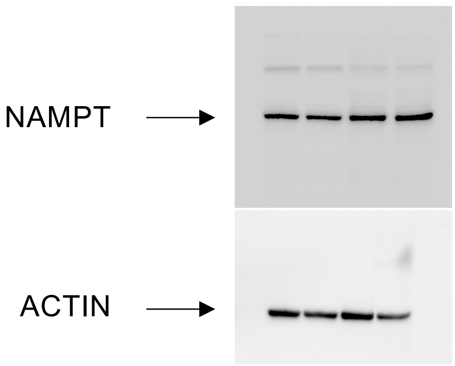


Figure 4C

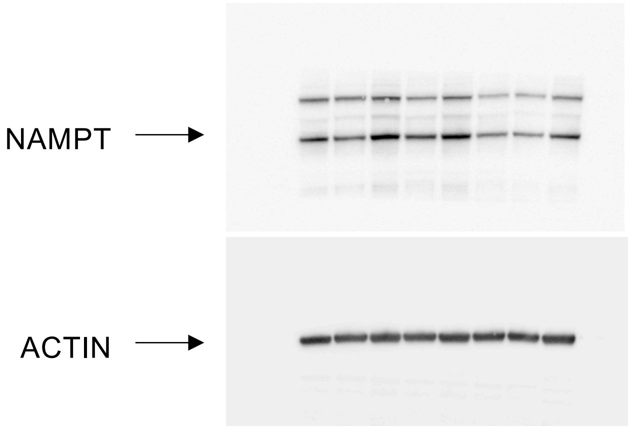


Figure 6A

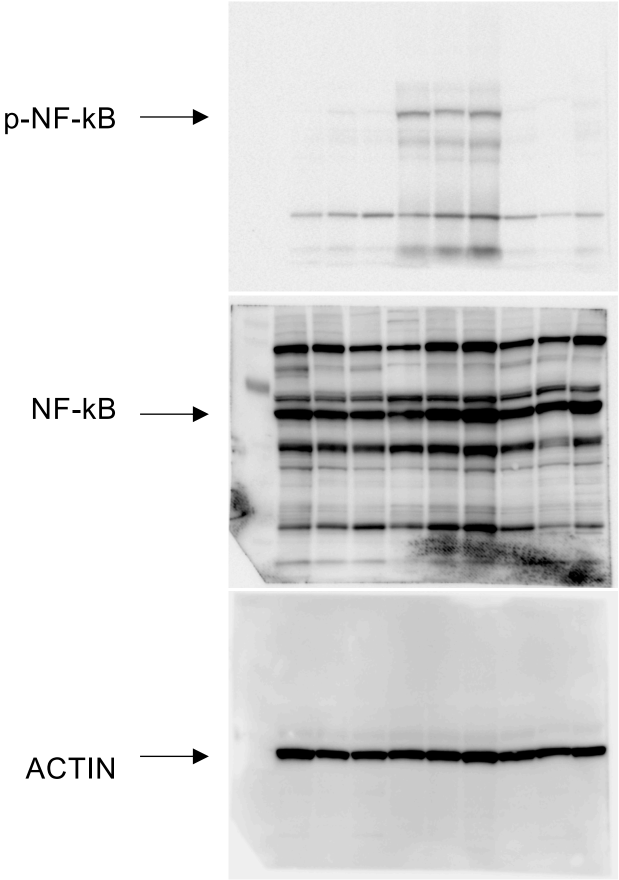


Figure 6C

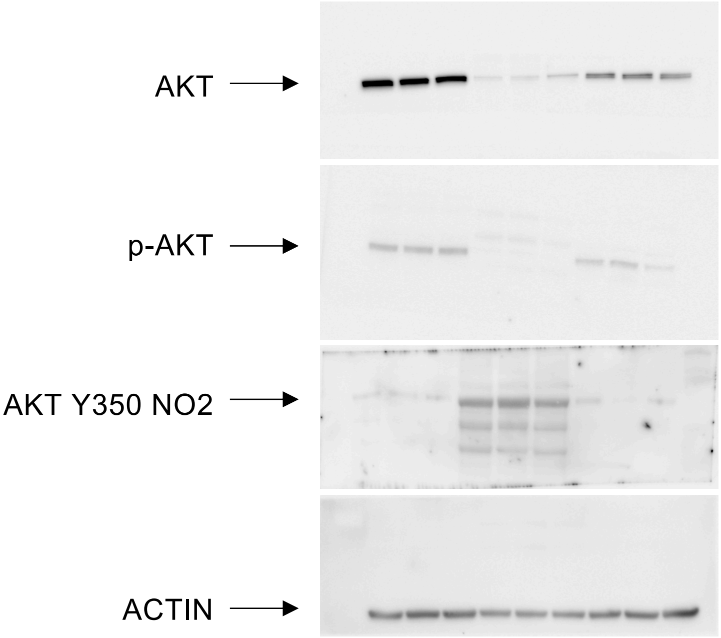

Figure 7A

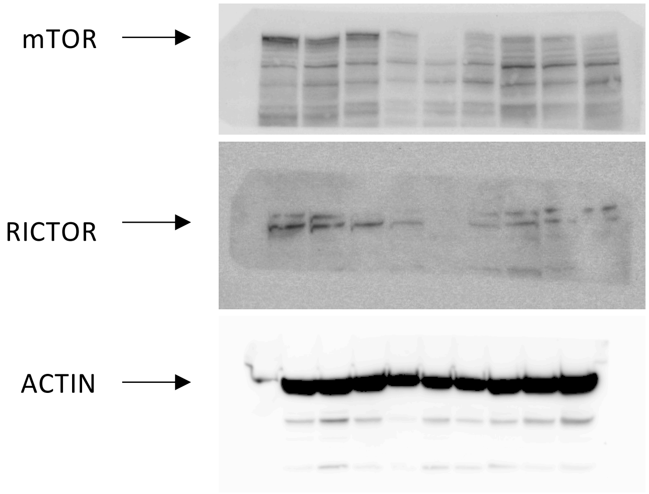


Figure 7B

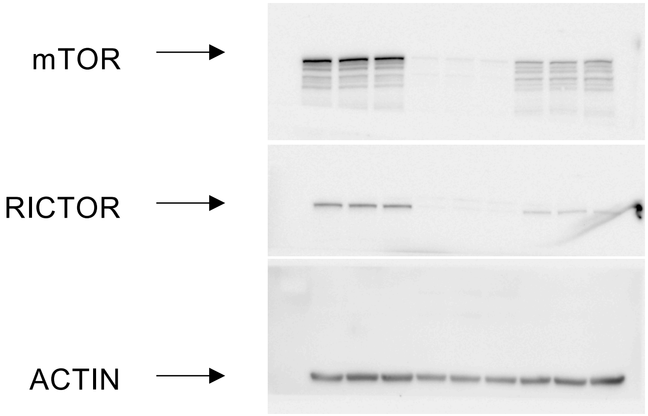


Figure 7C

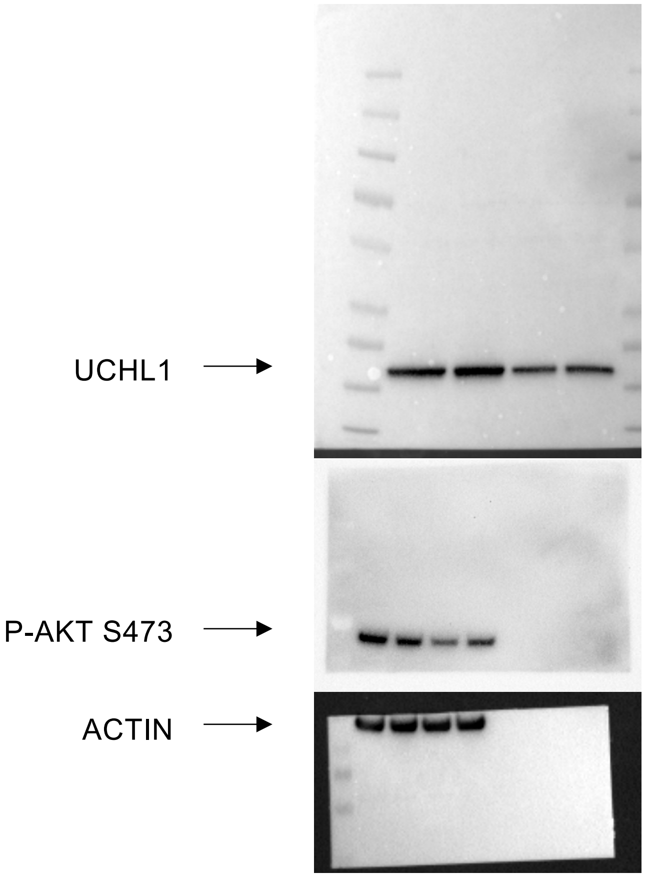


Figure 7 D

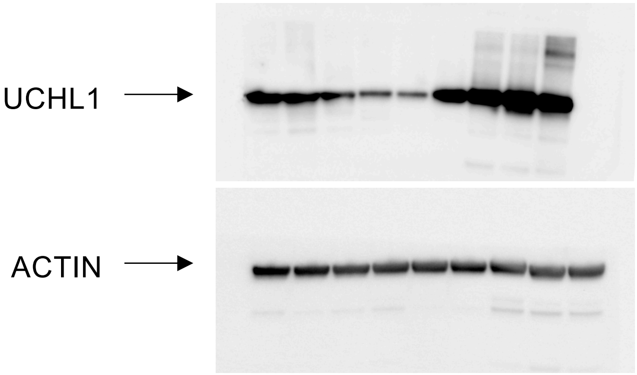

Supplement: Supplementary file 1 — Supplementary Information. [file 41598_2021_4444_MOESM1_ESM.pdf]
